# Infant Botulism in Costa Rica: A Case Report Featuring Electrodiagnostic Studies

**DOI:** 10.7759/cureus.110290

**Published:** 2026-06-05

**Authors:** Anthony Hong, Ana B Santos, Daniela Poveda, Alfonso Gutiérrez-Mata, Adriana Ulate-Campos

**Affiliations:** 1 Faculty of Medicine, University of Costa Rica, San José, CRI; 2 Neurology, Hospital Nacional de Niños “Dr. Carlos Sáez Herrera”, San José, CRI; 3 Pediatric Neurology, Hospital Nacional de Niños “Dr. Carlos Sáez Herrera”, San José, CRI

**Keywords:** botulism, clostridium botulinum, costa rica, electrodiagnostic studies, pediatric infectious disease

## Abstract

Infant botulism (IB) is a life-threatening neuroparalytic condition caused by a neurotoxin produced by *Clostridium botulinum (C. botulinum)*. We present the case of a four-month-old Costa Rican female who was hospitalized with poor sucking, feeding difficulties, constipation, hypotonia, and respiratory failure. Clinical history, physical examination findings, and electrodiagnostic studies strongly suggested IB. Although clinical suspicion primarily depends on a thorough history and physical examination, confirmatory tests can take considerable time to yield results. Electrodiagnostic studies may facilitate an earlier diagnosis and prompt initiation of treatment. Additionally, although this is only the second reported case in Costa Rica, it is the first in which electrodiagnostic studies were performed locally to support the diagnosis.

## Introduction

Infant botulism (IB) is a life-threatening neuroparalytic disorder affecting infants younger than one year of age. It is caused by colonization of the immature infant intestine by *Clostridium botulinum (C. botulinum)* and subsequent production of botulinum toxin [1]. C. botulinum is a spore-forming, Gram-positive bacillus commonly found in soil and aquatic sediments. IB occurs when clostridial spores are ingested and subsequently germinate in the gastrointestinal tract, releasing botulinum neurotoxin [2]. Botulinum neurotoxin binds irreversibly to presynaptic cholinergic nerve terminals, causing flaccid paralysis [1]. The first reported case of IB was described in 1976, and the only previously reported case in Costa Rica was published in 2014 [3]; however, electrodiagnostic studies were not performed. We report the second documented case of IB in Costa Rica and the first in which electrodiagnostic studies were performed to support the diagnosis.

## Case presentation

A four-month-old girl was brought to the emergency department of a secondary care clinic with a three-day history of progressive poor sucking, feeding difficulties, and irritability, along with a four-day history of constipation. She presented with respiratory distress, severe dehydration, minimal response to painful stimuli, generalized hypotonia, and dilated pupils. She was successfully intubated, and intravenous fluids were initiated. Following initial stabilization, she was transferred to our institution.

She had been born at term after an uncomplicated pregnancy and cesarean section delivery. Her birth weight was 3,190 g, and she had been exclusively breastfed. Newborn screening for inborn errors of metabolism was unremarkable, and her neurodevelopment had been normal. There was no history of honey or corn syrup consumption; however, she lived in an agricultural area and had been exposed to dust generated while her father was working with ceramic tiles inside the home two days before symptom onset.

On admission, her weight was 7.5 kg, temperature was 36 °C, oxygen saturation was 100%, heart rate was 120 beats/minute, respiratory rate was 20 breaths/minute under assisted mechanical ventilation, and blood pressure was 104/76 mmHg. On physical examination, she appeared adequately hydrated, with mydriatic pupils and absent direct and consensual light reflexes, absent gag reflex, absent vestibulo-ocular reflex, decreased facial expression, absent superficial abdominal reflexes, minimal response to painful stimuli, no pathologic posturing, generalized hypotonia and hyporeflexia, and absent Babinski and Hoffman signs.

Laboratory findings on admission are summarized in Table 1. Hemoglobin level was at the lower limit of normal for age, while leukocyte count was within normal limits. Serum biochemistry, including lactate, ammonia, and procalcitonin levels, was unremarkable. Urinary metabolic screening and toxicology screening were negative. Cerebrospinal fluid (CSF) biochemistry and cellularity were within normal limits. Blood, urine, CSF, and bronchoalveolar lavage cultures were negative. Head CT with and without contrast showed no abnormalities (Figure 1).

**Table 1:** Laboratory findings on admission

Laboratory test	Patient value	Reference range
Hemoglobin	10.1 g/dL	9.5–13.5 g/dL
Leukocyte count	6,360/mm³	5,000–19,500/mm³
Neutrophils	46%	15–45%
Lymphocytes	45%	45–75%
Serum ammonia	53 µmol/L	15–60 µmol/L
Lactate	0.9 mmol/L	0.5–2.2 mmol/L
Procalcitonin	0.09 ng/mL	<0.5 ng/mL
Vitamin B12	386 pg/mL	200–900 pg/mL

**Figure 1:**
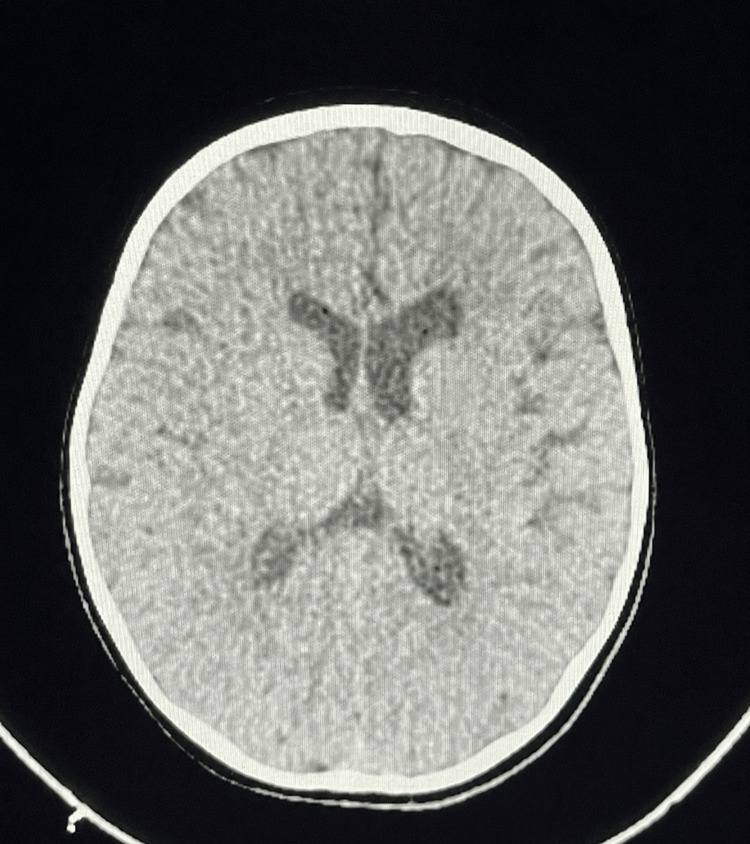
Non-contrast axial head CT showing no acute intracranial abnormalities and normal ventricular system CT: computed tomography

Neurology consultation raised a strong suspicion for IB; however, Human Botulism Immune Globulin Intravenous (BIG-IV) was not administered because it is currently unavailable in Costa Rica. Electroencephalography (EEG) was normal. Motor nerve conduction studies showed reduced compound motor action potential (CMAP) amplitudes in all examined motor nerves (Figure 2). Sensory nerve conduction studies were normal. Repetitive nerve stimulation (RNS) at 50 Hz using 10 stimuli demonstrated a 45% increase in CMAP amplitude, consistent with post-tetanic facilitation (Figure 3). Needle electromyography (EMG) showed spontaneous activity at rest and motor unit potentials with low amplitude, short duration, and early recruitment (Figure 4). These electrophysiological findings were consistent with a presynaptic neuromuscular junction disorder.

**Figure 2:**
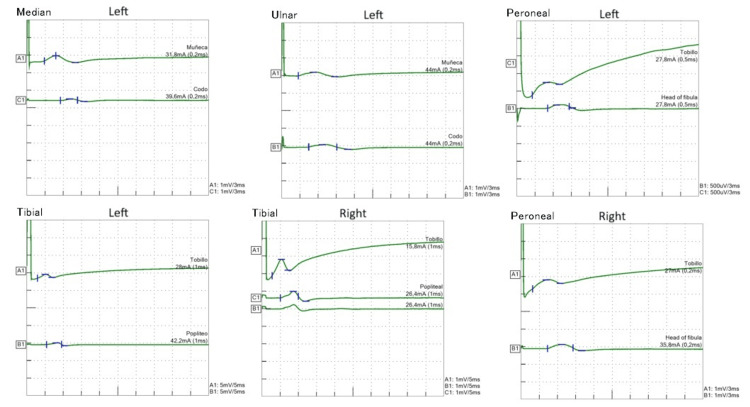
Motor neurography showing diminished CMAP in all of the nerves explored CMAP: compound motor action potential

**Figure 3:**
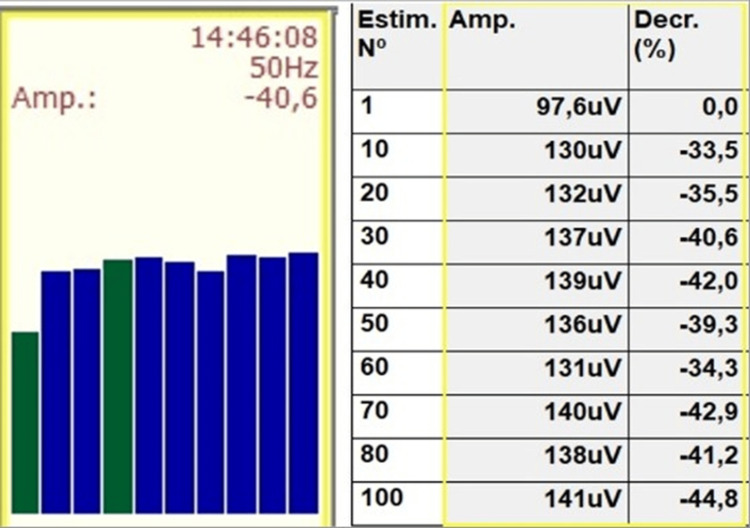
RNS test at 50 Hz showing 45% amplitude increase, in relation to post-tetanic potentiation RNS: repetitive nerve stimulation

**Figure 4:**
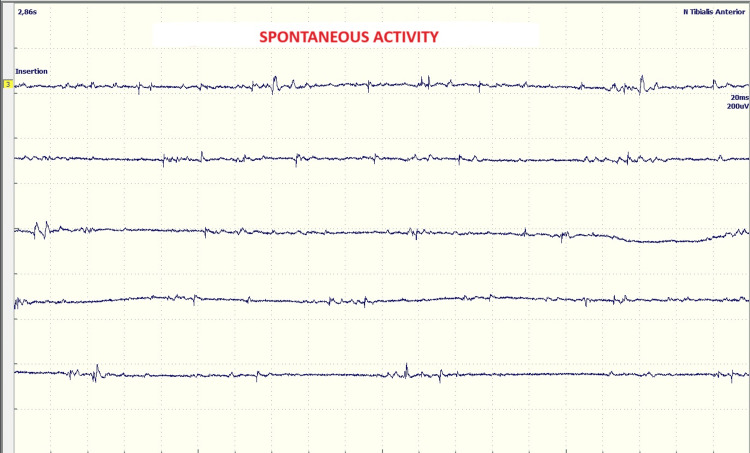
Needle EMG at the left tibialis anterior muscle showing spontaneous activity at rest and motor unit potentials with low amplitude and fast recruitment EMG: electromyography

Unfortunately, stool samples for *C. botulinum* were not obtained. Serum testing using the mouse bioassay neutralization method was negative for botulinum toxin. The patient required six days of management in the emergency department, followed by 11 days in the ICU and an additional 10 days of hospitalization. Supportive care remained the cornerstone of treatment. She received intravenous acyclovir and cefotaxime for six days until encephalitis and other infectious etiologies were excluded, and required a tracheostomy for prolonged airway support.

At the two-month follow-up visit, the patient had fully recovered from her neurological impairments, with normal feeding, muscle tone, strength, and neurological examination findings. No follow-up electrophysiological studies or neuroimaging were performed because of her complete clinical recovery. The family received counseling regarding environmental hygiene measures, including reducing infant exposure to dust and soil-contaminated environments, maintaining household cleanliness, and avoiding honey consumption during the first year of life.

## Discussion

The most frequently recognized source of *C. botulinum* spores is honey, as contaminated honey consumption has been implicated in several cases of IB [1]. However, *C. botulinum* spores have been identified in less than 25% of honey samples, and only a minority of patients with botulism report a history of honey consumption [4,5]. Therefore, additional environmental sources may directly expose infants to *C. botulinum*. In the present case, there was no history of honey consumption; however, the patient was likely exposed to ceramic dust. Exposure to soil and dust from active construction sites has been associated with an increased risk of IB due to the presence of *C. botulinum* spores in environmental dust [5].

IB presents with a wide spectrum of clinical manifestations; however, in this case, the diagnosis was suspected early based on the initial symptoms. Constipation is often an early sign of IB, attributed to the effect of the toxin on the intestinal neuromuscular junction [1]. This is typically followed by lethargy and poor feeding, and subsequently by symmetric descending hypotonia and weakness [2], as observed in our patient. The differential diagnosis of IB is broad and includes conditions such as spinal muscular atrophy and Guillain-Barré syndrome. Spinal muscular atrophy was considered unlikely due to the absence of a chronic history of hypotonia. Guillain-Barré syndrome was also considered less likely given the absence of preceding gastrointestinal or respiratory infection, together with the neurophysiological findings. Additionally, 88% of IB cases occur in infants ≤6 months of age, with a mean age of onset of 3.8 ± 2.2 months, whereas Guillain-Barré syndrome is more common in older children [5,6]. 

Diagnostic criteria for IB have been previously proposed and are divided into five domains (A-E): clinical features, brain and spinal imaging, cerebrospinal fluid analysis, electrodiagnostic studies, and laboratory confirmation from clinical specimens [7]. Our patient fulfilled most of these criteria, except for laboratory confirmation. The gold standard for toxin detection remains the mouse bioassay and neutralization test [4]; however, this case highlights the diagnostic value of electrodiagnostic studies. Moreover, toxin detection is less frequent in serum than in stool, with only approximately 1% of infant cases in the United States showing serum positivity [8]. 

According to proposed diagnostic algorithms, infants presenting with acute descending weakness and autonomic signs (A1 and A2 criteria), in the absence of an alternative explanation for acute flaccid paralysis, should undergo needle EMG to evaluate neuromuscular transmission. The following electrodiagnostic features support the diagnosis of IB [7]: D1. Motor nerve conduction studies: reduced CMAP amplitudes. D2. Sensory nerve conduction studies: normal sensory nerve action potentials (SNAPs). D3. Needle EMG: short-duration, low-amplitude motor unit potentials with a myopathic-like pattern and possible denervation activity. D4. RNS: 50 Hz tetanic stimulation demonstrating an incremental CMAP response compared with baseline. D5. Single-fiber EMG: increased jitter consistent with neuromuscular junction dysfunction, improving with higher-frequency stimulation. Our patient met all of these criteria except for D5, which was not performed.

The sensitivity and specificity of EMG, RNS, and nerve conduction studies in IB remain undetermined; however, these studies are valuable in differentiating IB from other neuromuscular disorders such as Guillain-Barré syndrome and myasthenia gravis. This distinction is particularly important in sporadic cases, as it directly impacts urgent therapeutic decisions, including administration of antitoxin in suspected IB versus immunoglobulin or plasmapheresis in Guillain-Barré syndrome. Importantly, electrodiagnostic abnormalities may persist for weeks after symptom onset, even when the circulating toxin is no longer detectable in serum [9].

Gutiérrez et al. proposed a diagnostic protocol based on supramaximal nerve stimulation followed by 50 Hz tetanization for 10 seconds and repeated stimulation at 30-second intervals until baseline recovery [10]. They described diagnostic features including reduced CMAP amplitudes in at least two muscle groups, post-tetanic and tetanic facilitation exceeding 120% of baseline, prolonged facilitation beyond 120 seconds, and absence of post-tetanic exhaustion. Witoonpanich et al. evaluated 63 patients during an outbreak of foodborne botulism and reported post-exercise facilitation ranging from 40% to 200% following supramaximal stimulation [11]. They proposed a 25% increment cutoff, demonstrating 95% sensitivity and high specificity for diagnosis. For IB, a 10-second high-frequency repetitive nerve stimulation protocol was suggested as an alternative to voluntary contraction.

In our case, post-tetanic facilitation reached 45%, exceeding the proposed 25% cutoff, and showed a clear incremental response, similar to the case reported by Dileta et al. [7]. A shorter protocol using 10 stimuli was selected instead of longer stimulation paradigms due to patient discomfort; however, a longer stimulation period may have resulted in an even greater facilitation response. Similarly, Cornblath et al. reported that most infants with IB demonstrate abnormal spontaneous activity on EMG, including fibrillation potentials and positive sharp waves, as well as low-amplitude CMAPs and motor unit potentials [12]. Our patient achieved full neurological recovery and remained asymptomatic at the last follow-up visit, consistent with the expected clinical course of IB.

## Conclusions

IB is a rare but potentially life-threatening neurologic disorder that should be considered in infants presenting with hypotonia, poor feeding, constipation, and autonomic dysfunction. Early clinical recognition is crucial when confirmatory laboratory testing is unavailable or delayed. This report highlights the value of electrodiagnostic studies in supporting the diagnosis and guiding timely management. The patient achieved complete neurological recovery with supportive care, underscoring the favorable prognosis associated with early recognition and appropriate management.
